# *In vitro* studies to investigate the potential neuroprotective and neurotransmitter modulation effects of a standardized *Ginkgo biloba* extract associated with phosphatidylserine

**DOI:** 10.3389/fnut.2026.1764334

**Published:** 2026-02-18

**Authors:** Mehtap Kara, Gozde Hasbal-Celikok, Pilar Gómez-Serranillos, Marta Sánchez Gómez-Serranillos, Claudia Owsianik, Tugba Yilmaz-Ozden, Ezgi Öztas, Nazli Arda, Merve Tunc, Çiğdem Sevim, Giovanna Petrangolini, Fazle Rabbani, Ikram Ujjan, Amjad Khan

**Affiliations:** 1 Department of Pharmaceutical Toxicology, Faculty of Pharmacy, Istanbul University, Istanbul, Türkiye; 2 Department of Biochemistry, Faculty of Pharmacy, Istanbul University, Istanbul, Türkiye; 3 Department of Pharmacology, Pharmacognosy and Botany, Faculty of Pharmacy, Complutense University of Madrid, Madrid, Spain; 4 Department of Molecular Biology and Genetics, Faculty of Science, Istanbul University, Istanbul, Türkiye; 5 Department of Molecular Biology and Genetics, Institute of Graduate Studies in Sciences, Istanbul University, Istanbul, Türkiye; 6 Department of Medical Pharmacology, Medicine Faculty, Kastamonu University, Kastamonu, Türkiye; 7 Medical Department, Indena S.p.A, Milan, Italy; 8 Department of Psychiatry, Lady Reading Hospital (LRH), Peshawar, Pakistan; 9 Department of Pathology, Liaquat University of Medical and Health Sciences, Jamshoro, Pakistan; 10 Department of Biochemistry, Liaquat University of Medical and Health Sciences, Jamshoro, Pakistan; 11 Nuffield Division of Clinical Laboratory Sciences, University of Oxford, Oxford, United Kingdom

**Keywords:** BDNF, cognition, emotional well-being, *Ginkgo biloba*, neurotransmitter modulation, nutraceuticals, oxidative stress, phosphatidylserine

## Abstract

Cognitive impairment and mood disturbances are increasingly linked to underlying mechanisms such as oxidative stress, neurotransmitter dysregulation, and reduced neurotrophic support. As conventional pharmacological treatments often provide limited efficacy or are associated with tolerability concerns, there is growing scientific interest in botanical supporting strategies that may modulate the above pathways and provide complementary support for cognitive function and emotional well-being. This study aimed to investigate the mechanistic basis of a botanical association consisting of a standardized *Ginkgo biloba* extract (GBE) from leaves and phosphatidylserine (PS) (combined referred as GBP) (Virtiva™ Plus), focusing on its potential effects on neurotransmitter-related enzymes and receptors, neuroprotection under oxidative stress, neurotrophic signaling, and antioxidant capacity. GBP was characterized analytically and evaluated in a series of validated *in vitro* assays using human SH-SY5Y neuroblastoma cells and multiple cell-free antioxidant systems. Neurotransmitter effect assays demonstrated that GBP inhibited acetylcholinesterase (AChE) and monoamine oxidase-A (MAO-A) in a concentration-dependent manner, suggesting selective modulation of cholinergic and monoaminergic pathways relevant to cognition and mood regulation. Enzyme modulation observed at micromolar concentrations supports mechanistic plausibility of *G. biloba* constituents in neurochemical pathways rather than direct modeling of physiological exposure. In SH-SY5Y cells exposed to hydrogen peroxide (H_2_O_2_), GBP improved cell viability, confirming no intrinsic cytotoxicity, and reduced lactate dehydrogenase (LDH) release, indicating protection against oxidative stress-induced cytotoxicity. GBP also partially restored brain-derived neurotrophic factor (BDNF) levels in SH-SY5Y cells suppressed by H_2_O_2_, supporting preservation of neurotrophic signaling linked to neuronal survival and synaptic plasticity. In cell-free antioxidant assays, GBP demonstrated broad-spectrum activity across 2,2-Diphenyl-1-picrylhydrazyl (DPPH), 2,2′-azino-bis(3-ethylbenzothiazoline-6-sulfonic acid) (ABTS), Ferric reducing antioxidant power (FRAP), Oxygen radical absorbance capacity (ORAC), Hydroxyl radical antioxidant capacity (HORAC), total phenolic content (TPC), and total antioxidant status (TAS) assays, validating its capacity to neutralize free radicals and support redox balance. Collectively, these findings provide mechanistic evidence supporting the biological plausibility of multi-target actions of GBP, including neurotransmitter modulation, antioxidant effects, neuroprotection, and preservation of neurotrophic signaling, which may help explain previously reported cognitive- and mood-related outcomes.

## 1 Introduction

Cognitive impairment and mood disturbances are increasingly recognized as major contributors to reduced quality of life and productivity across populations. These conditions arise from complex and interacting biological pathways, including oxidative stress, neurotransmitter imbalance, mitochondrial dysfunction, and neuro-inflammation, each of which can disrupt normal neural signaling and cognitive processing. Although a range of pharmacological treatments, such as antidepressants, anxiolytics, and cognition-enhancing agents, are available, their effectiveness is often constrained by several limitations. Many therapies yield only modest symptomatic improvement, require prolonged treatment periods before benefits emerge, and may produce undesirable side effects that reduce adherence. In addition, access to specialized psychiatric or neurological care can be limited in many regions, further diminishing the reach of conventional interventions. These challenges have contributed to rising interest in complementary, nutraceutical, and plant-derived strategies aimed at supporting neurocognitive function and emotional well-being (1–4).

Botanical ingredients have been widely investigated for their potential benefits in cognitive function, mood regulation, and overall emotional well-being. Among these, *Ginkgo biloba* (GB) has attracted substantial scientific attention for health benefits due to its worldwide long-standing use in traditional medicine and a steadily expanding body of modern recent pharmacological evidence supporting its potential role in cognitive performance and emotional well-being (5–19). GB has a particularly long history of traditional use in East Asia, especially in China, Japan, and Korea, where its leaves and seeds have been incorporated into medicinal practices for centuries. Today, the plant is cultivated across Europe, the Americas, and other regions, and its standardized extracts are used in botanical preparations for neurocognitive and emotional support.

A growing body of clinical and preclinical evidence supports the potential efficacy of standardized *G. biloba extract* (GBE) in enhancing memory, executive function, and mood in both healthy individuals and those with mild cognitive or mood disturbances. Extracts derived from the leaves of GB are among the most extensively studied botanical preparations and have been evaluated for their potential to support cognitive performance and to contribute to the management of age-related neurodegenerative conditions, including Alzheimer’s disease (AD), Parkinson’s disease (PD), and dementia (7, 12–15).

The potential health benefits of GBE are largely attributed to its diverse bioactive compounds, including terpenoids, flavonoids, polyphenols, and organic acids (6, 20). GBE is particularly rich in bilobalide, ginkgolides (A, B, and C), and flavone glycosides such as quercetin, isorhamnetin, and kaempferol. These constituents have been associated with antioxidant, anti-inflammatory, neuroprotective, and anti-apoptotic properties, which may act additively or synergistically to mitigate oxidative stress, modulate neurotransmitter systems, and improve cerebral blood flow, thereby supporting cognitive function and emotional well-being (6–9, 12, 17, 19, 20).

Alongside GBE, a considerable body of clinical and mechanistic evidence also suggests the potential of phosphatidylserine (PS) in supporting brain health (21–31). PS is an essential phospholipid and a key structural component of neuronal membranes, where it contributes to membrane fluidity, receptor dynamics, intracellular signaling, and neurotransmitter release. Supplementation with PS has been associated with improvements in memory, attention, and overall cognitive performance, and its role in maintaining synaptic integrity, modulating stress responses and supporting cholinergic and dopaminergic pathways further underscores its relevance to both cognitive function and emotional well-being.

Given the potential for additive or synergistic effects, there is increasing scientific interest in the association of GBE and PS for cognitive health and emotional well-being. In addition to its intrinsic neuroprotective and cognition-supporting properties, PS may exert a complementary role by optimizing the interaction of bioactive compounds into neuronal membranes (28). This dual action provides a clear rationale for formulating GBE and PS together to optimize outcomes related to cognitive performance and emotional well-being. Previous clinical findings have reported promising preliminary outcomes for this innovative association (27, 32).

In the present study, we investigated the mechanistic basis of the association of a standardized GBE and PS (hereafter referred to as GBP). We assessed its potential antioxidant and neuroprotective properties in both neuronal cellular and cell-free systems, along with its effects on key neurotransmitter-related pathways. These analyses aim to provide supportive evidence for the potential of this GBP association as an effective natural approach for promoting cognitive function and emotional well-being.

## 2 Materials and methods

### 2.1 Botanical ingredients

The standardized GBP preparation used in this study was composed of ~ 25% GBE (from leaves) and 75% sunflower-derived lecithin standardized to contain 20% PS, corresponding to a final composition of ≥5% ginkgoflavonglycosides, ≥0.5% ginkgoterpenes, and ≥12% PS, as verified by High-Performance Liquid Chromatography (HPLC) analysis (Virtiva™ Plus, Indena S.p.A., Milan, Italy) (27, 32).

### 2.2 *In vitro* assays

A comprehensive panel of *in vitro* assays was conducted to characterize the neurotransmitter-modulating, neuroprotective, and antioxidant properties of the standardized GBP preparation. SH-SY5Y human neuroblastoma cells were selected as a well-established *in vitro* neuronal model generally used to investigate oxidative stress–induced neurotoxicity, neuroprotection, and neurotransmitter-related mechanisms relevant to cognitive and mood-related processes. The assays were selected to evaluate key mechanisms relevant to cognitive function and emotional well-being, including modulation of neurotransmitter-related enzymes and receptors, protection against oxidative stress, maintenance of neurotrophic signaling, and direct antioxidant activity. The concentration ranges used in the assays were selected based on preliminary screening experiments and on ranges commonly employed in published mechanistic *in vitro* studies of botanical extracts to explore enzyme-ligand interactions (33–35). These biological processes collectively contribute to neuronal function, resilience, and survival. All experiments were performed according to validated methodologies previously employed in our laboratory for the assessment of botanical extracts, with minor adaptations for the current formulation (33–35), and were conducted under controlled laboratory conditions to ensure data reliability and reproducibility. Unless otherwise stated, all concentrations reported for enzymatic and receptor-binding assays refer to final concentrations in the assay mixture.

#### 2.2.1 Neurotransmitter-related enzymatic assays

To investigate potential effects on neurotransmission, GBP was evaluated across several key enzymatic and receptor targets involved in cholinergic, monoaminergic, and GABAergic regulation. Acetylcholinesterase (AChE) and monoamine oxidase-A (MAO-A) were assessed to determine whether GBP influences pathways relevant to cognition and mood. In addition, γ-aminobutyric acid transaminase (GABA-T) was examined to evaluate potential effects on inhibitory neurotransmission. Possible interactions with the γ-Aminobutyric acid sub-type A (GABA-A) receptor - the major inhibitory ligand-gated ion channel in the central nervous system - were assessed through [^3^H]-muscimol displacement. All enzymatic and receptor-binding assays were performed according to established protocols (33–35).

#### 2.2.2 Neuroprotection effect on SH-SY5Y cells under oxidative stress

Potential neuroprotective effects of GBP were evaluated in human SH-SY5Y neuroblastoma cells exposed to H_2_O_2_ as a model of oxidative injury. Cell viability was measured using the 3-(4,5-dimethylthiazol-2-yl)-2,5-diphenyltetrazolium bromide (MTT) assay, and membrane integrity was assessed by lactate dehydrogenase (LDH) release, following validated procedures (33–35).

#### 2.2.3 Brain-derived neurotrophic factor (BDNF) expression levels under oxidative stress

To evaluate the GBP potential effects on neurotrophic signaling, Brain-derived neurotrophic factor (BDNF), a key mediator of neuronal survival and synaptic plasticity, was quantified in SH-SY5Y cells in the presence of H_2_O_2_-induced oxidative stress using established methods (35).

#### 2.2.4 Cytotoxicity assessment in SH-SY5Y cells

Intrinsic cytotoxicity of GBP was evaluated by exposing SH-SY5Y cells to increasing concentrations of GBP under standard culture conditions. Cell viability was assessed using the MTT assay as described previously (33–35).

#### 2.2.5 Antioxidant effects in cell-free systems

The direct potential antioxidant activity of GBP was examined using a panel of complementary chemical assays, including 2,2-Diphenyl-1-picrylhydrazyl (DPPH) and 2,2′-azino-bis(3-ethylbenzothiazoline-6-sulfonic acid) (ABTS) radical-scavenging activity, Ferric reducing antioxidant power (FRAP), Oxygen radical absorbance capacity (ORAC), Hydroxyl radical antioxidant capacity (HORAC), Total Phenolic Content (TPC), and Total Antioxidant Status (TAS). All assays were carried out according to validated analytical methods (33–35).

### 2.3 Statistical analysis

GBP potential activity parameter results were expressed as mean ± standard deviation (SD) from three independent experiments. Statistical analyses were performed using one-way analysis of variance (ANOVA) followed by Dunnett’s *post hoc* test in SPSS (Statistical Package for the Social Sciences) software (version 20; IBM SPSS Inc., New York, NY, United States). A *p*-value of less than 0.05 was considered statistically significant.

## 3 Results

### 3.1 Neurotransmitter enzymatic assays

**Figure 1:**
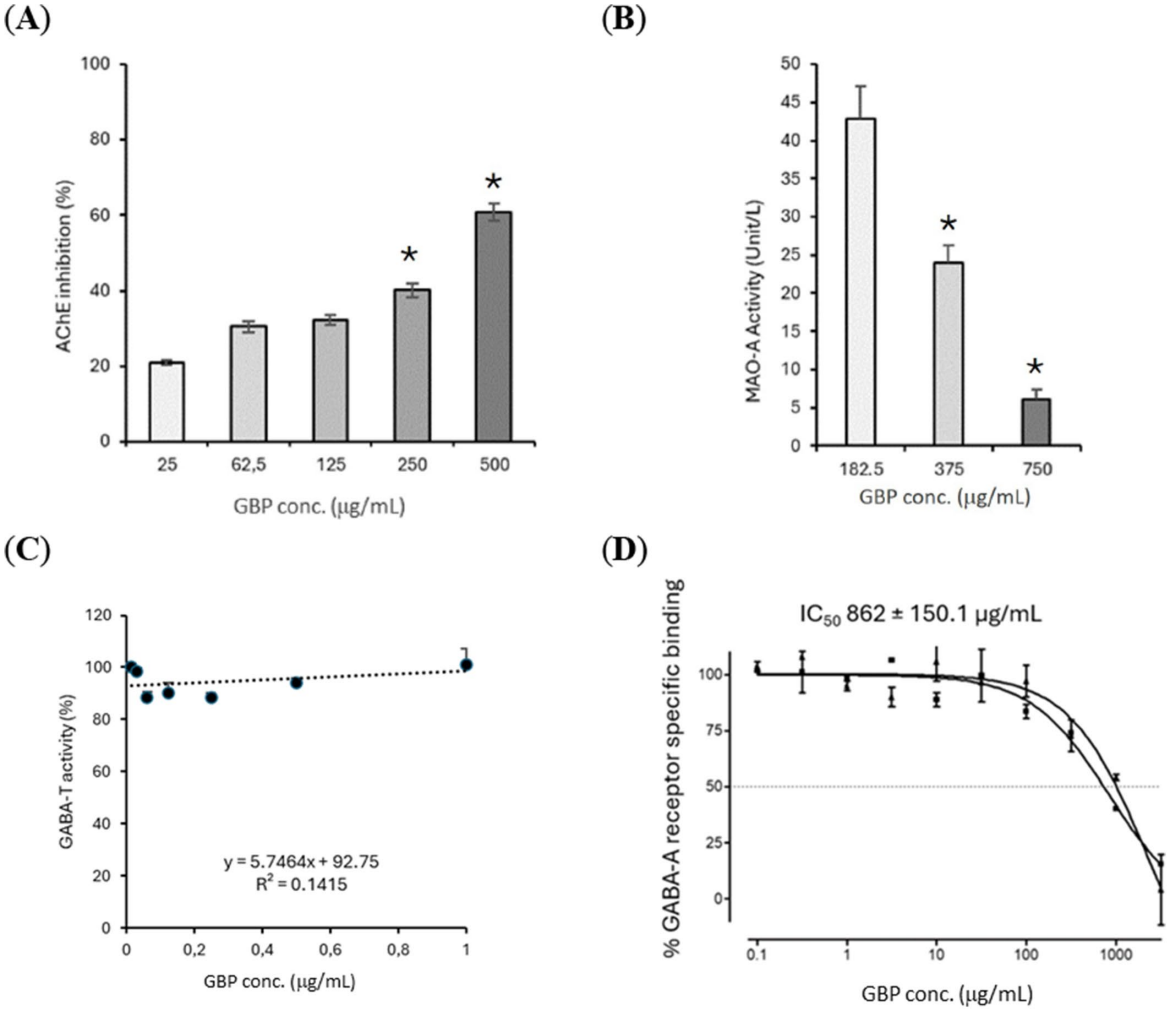
*In vitro* assays showing the effects of the association of GBE from leaves and PS, combined as GBP, on neurotransmitter-related targets. **(A)** AChE inhibition. **(B)** MAO-A inhibition. **(C)** GABA-T inhibition **(D)** GABA-A receptor specific binding. Results are presented as mean ± SEM (*n* = 3 independent experiments). Error bars represent standard error of the mean. ^*^p < 0.05, indicating statistically significant differences. SEM, Standard error of the mean.

GBP modulated several neurotransmission-related targets *in vitro*. AChE activity was inhibited in a concentration-dependent manner across the 25–500 μg/mL range (Figure 1A). MAO-A activity was also reduced in a dose-dependent fashion at concentrations between 182.5 and 750 μg/mL (Figure 1B). By contrast, GABA-T activity remained largely unchanged across the tested concentrations (0.015–1 μg/mL) (Figure 1C), while GABA-A receptor binding showed weak but measurable displacement of [^3^H]-muscimol at higher concentrations (0.01–1,000 μg/mL), yielding an IC_50_ of 862 ± 150.1 μg/mL (Figure 1D). These findings suggest that GBP may influence neurotransmission primarily through cholinergic and monoaminergic pathways, mechanisms associated with memory, attention, and mood regulation, while exerting minimal direct effects on GABAergic signaling.

### 3.2 Neuroprotection effect on SH-SY5Y cells under oxidative stress

**Figure 2:**
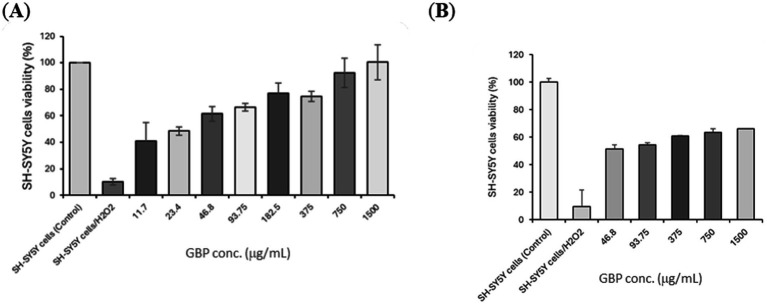
Effects of GBP on SH-SY5Y cells under H_2_O_2_-induced oxidative stress. **(A)** Results of cell viability assessed by MTT assay following treatment with H_2_O_2_ and increasing concentrations of GBP. **(B)** Membrane integrity evaluated by LDH release under the same conditions. GBP, *G. biloba*-PS association.

H_2_O_2_ exposure markedly reduced SH-SY5Y cell viability, as shown by both the MTT and LDH assays (Figure 2). GBP treatment improved cell survival in a concentration-dependent manner in the MTT assay across 11.7–1,500 μg/mL (Figure 2A).

Similarly, GBP decreased LDH release at 46.8–1,500 μg/mL (Figure 2B), reflecting reduced membrane damage and consistent with improved overall cell viability under oxidative stress.

These results indicate that GBP may help maintain neuronal integrity during oxidative challenge, suggesting a protective effect likely mediated through mechanisms that enhance cellular resilience and limit oxidative damage.

### 3.3 Brain-derived neurotrophic factor (BDNF) expression levels under oxidative stress

**Figure 3:**
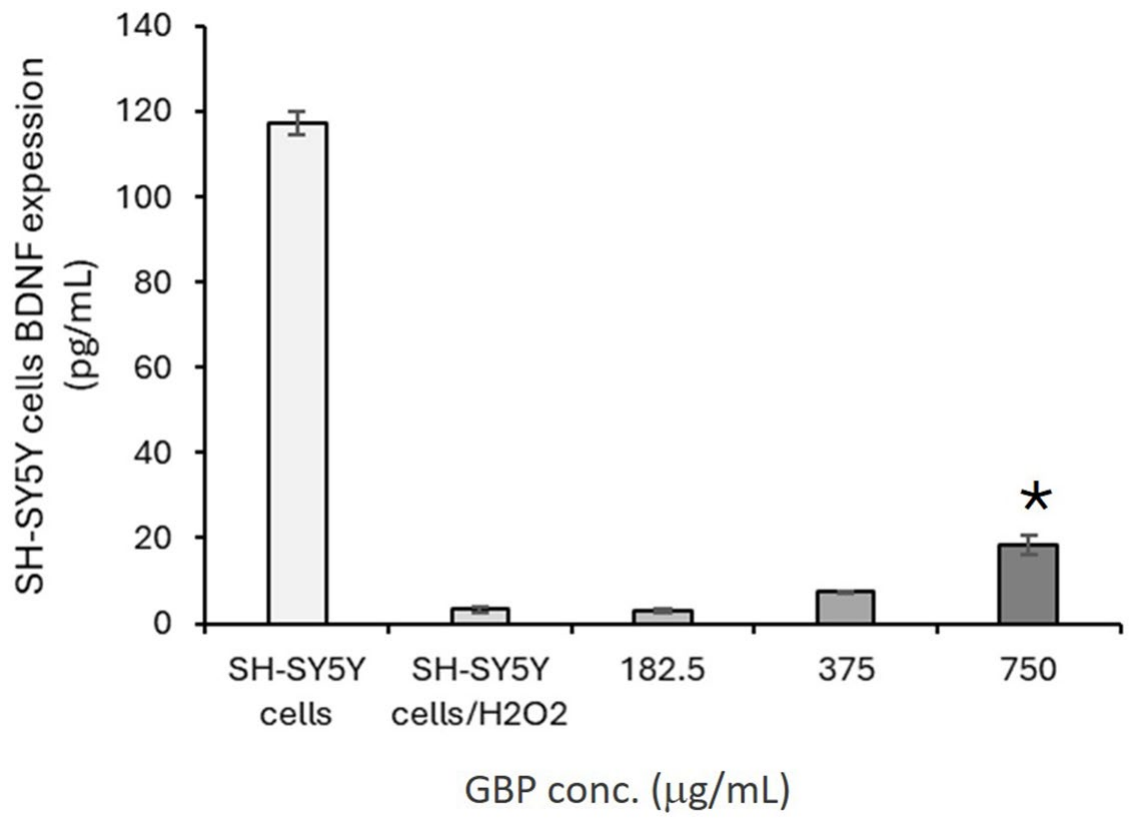
Effects of GBP on BDNF expression in SH-SY5Y cells exposed to H_2_O_2_-induced oxidative stress. ^*^p < 0.05 vs. H_2_O_2_-treated control, indicating statistically significant differences.

Exposure of SH-SY5Y cells to H_2_O_2_ significantly decreased BDNF expression levels, confirming the suppressive effect of oxidative stress on neurotrophic signaling. Treatment with GBP (182.5–750 μg/mL) partially restored BDNF levels, indicating a protective effect on neurotrophic support under oxidative stress conditions (Figure 3).

These findings suggest that GBP may help preserve neurotrophic pathways involved in synaptic plasticity and neuronal survival, supporting cellular mechanisms that are essential for cognitive function and stress resilience.

### 3.4 Cytotoxicity in SH-SY5Y cells

Intrinsic cytotoxicity of GBP was evaluated by exposing SH-SY5Y cells to increasing concentrations of the preparation under standard culture conditions. Cell viability remained unchanged across the tested concentration range when assessed by the MTT assay (data not shown), indicating that GBP did not exert cytotoxic effects under basal conditions. These results suggest that GBP was well tolerated by neuronal cells at the concentrations used in the mechanistic assays, supporting its suitability for further development.

### 3.5 GBP antioxidant effects in cell-free systems

**Figure 4:**
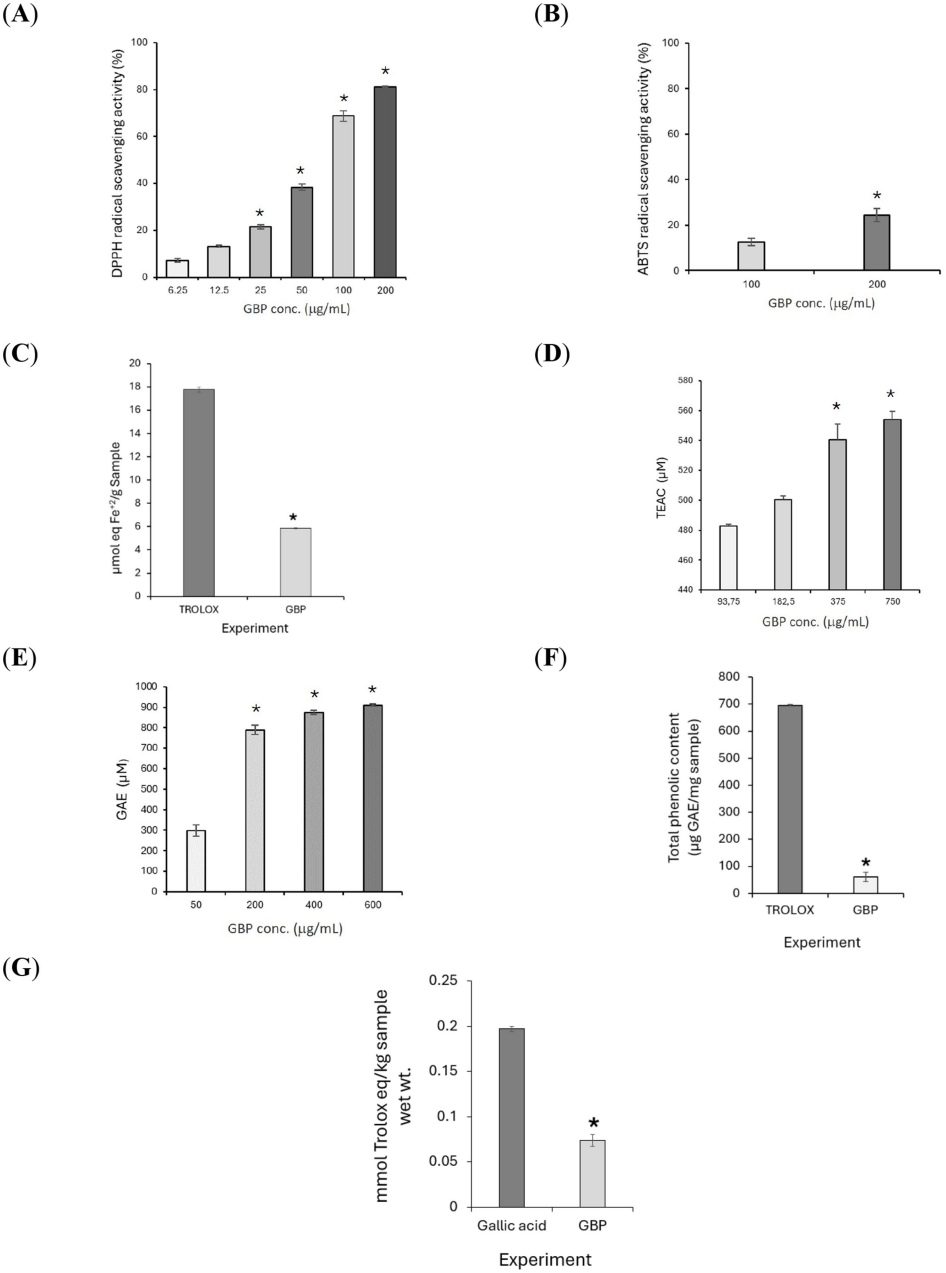
Antioxidant effect of GBP in cell-free chemical assays. **(A)** DPPH assay. **(B)** ABTS assay. **(C)** FRAP assay. **(D)** ORAC assay. **(E)** HORAC assay. **(F)** TPC Folin-Ciocalteau assay. **(G)** TAS assay. ^*^p < 0.05 vs. control, indicating statistically significant differences. TEAC, Trolox equivalent antioxidant capacity; GAE, Gallic acid equivalents.

GBP demonstrated broad-spectrum antioxidant activity across multiple chemical assay platforms. In the DPPH assay, GBP showed dose-dependent radical-scavenging activity within the 6.25–200 μg/mL range, with an EC_50_ of 70.05 ± 2.48 μg/mL (Figure 4A). Concentration-dependent ABTS^+^• quenching was also observed at 100–200 μg/mL (Figure 4B). In the FRAP assay, GBP displayed ferric-reducing power at 1 mg/mL, yielding 5.87 ± 0.036 μmol Fe^2+^ equivalents per gram of extract (Figure 4C). ORAC analysis revealed increasing peroxyl radical scavenging at 93.75–750 μg/mL (Figure 4D), while HORAC activity was observed between 50 and 600 μg/mL (Figure 4E). TPC was measured as 61.33 ± 17.00 μg gallic acid equivalents/mg (GAE/mg) extract (Figure 4F), and TAS was 0.074 ± 0.0065 mmol Trolox equivalents/kg (Figure 4G). Collectively, these results indicate that GBP may exert antioxidant effects through multiple mechanisms, including free radical scavenging and redox-modulating activity, which may contribute to its neuroprotective potential observed in cellular assays.

**Figure 5:**
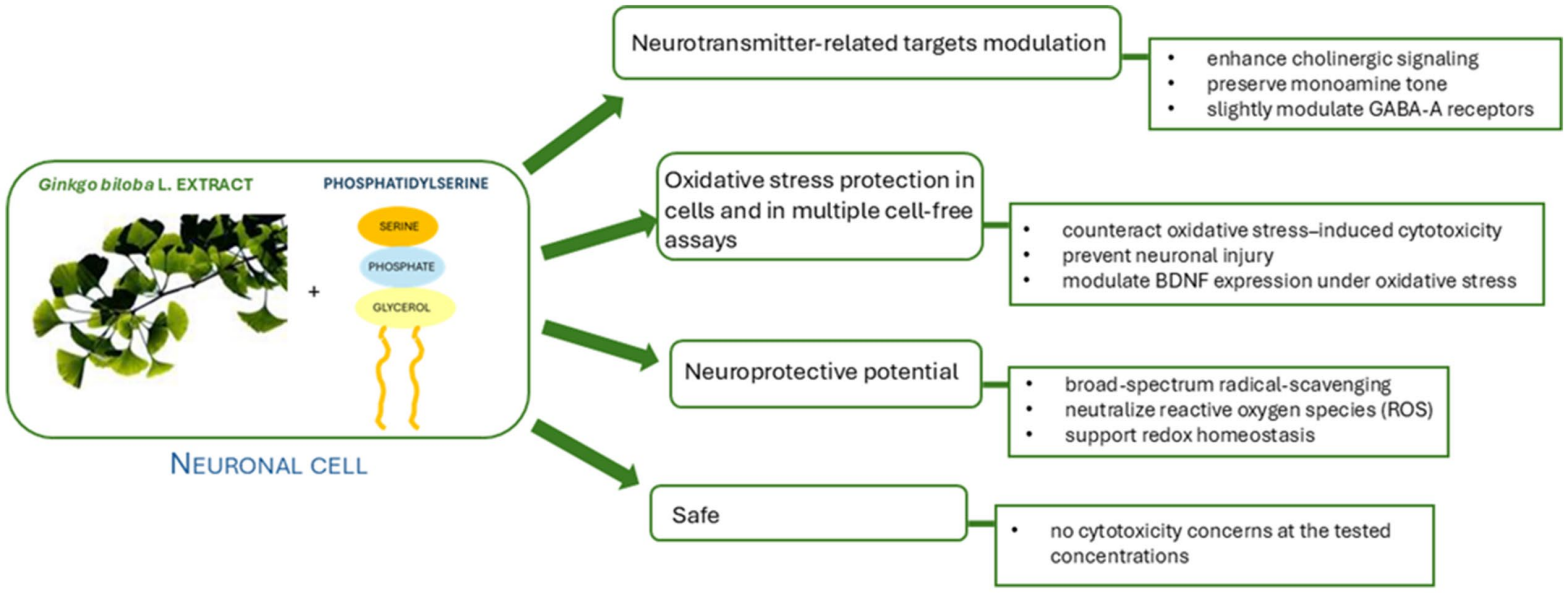
Schematic overview of the potential mechanistic actions observed for the GBP in the present *in vitro* study, including modulation of neurotransmission-related targets, protection against oxidative stress, antioxidant activity, and lack of cytotoxicity at tested concentrations.

Overall, these outcomes confirm that the GBP association may provide broad-spectrum antioxidant effects relevant to the prevention of oxidative stress–related cellular dysfunction. The mechanistic evidence supporting the potential beneficial effects of GBP on cognitive function and emotional well-being are summarized in Figure 5.

## 4 Discussion

The present *in vitro* studies demonstrate that the association of GBE and PS can potentially modulate neurotransmitter-related pathways and safely exert complementary antioxidant and neuroprotective effects, consistent with mechanisms implicated in cognitive function and emotional well-being. Importantly, GBP showed no intrinsic cytotoxicity up to the highest concentrations tested, supporting its safety in neuronal models. To our knowledge, this represents the first investigation of the supporting mechanistic evidence for this association, providing novel insights that complement existing clinical findings (27, 32).

Neurotransmitter modulation was a prominent finding in this study. GBP inhibited AChE and MAO-A enzymes in a concentration-dependent manner, suggesting potential to enhance cholinergic signaling and preserve monoamine tone—mechanisms directly relevant to memory, attention, and mood regulation. Importantly, the magnitude of AChE and MAO-A inhibition observed *in vitro* was modest when compared with potent pharmaceutical reference inhibitors, and is therefore more consistent with a supportive neuro-modulatory role rather than a drug-like pharmacological effect. Such moderate enzyme modulation may nonetheless contribute to cumulative functional benefits when combined with complementary mechanisms such as antioxidant protection and neurotrophic support. Previous studies have shown that GBEs inhibit both MAO-A and MAO-B activity (36), while PS specifically inhibits Monoamine oxidase B (MAO-B) (37). These independent findings provide biological plausibility for the monoaminergic effects observed here and highlight the potential complementarity of the GBE and PS within the formulation. By contrast, GABA-T activity was unaffected, and GABA-A receptor binding was weak and only evident at high concentrations. This observation is consistent with evidence that *Ginkgo*-derived terpene trilactones, such as ginkgolides and bilobalide, function as antagonists or negative modulators at GABA-A receptors (38, 39). Such interactions appear to be modest relative to their actions on cholinergic and monoaminergic systems and are more likely to contribute to the regulation of excitatory-inhibitory balance and neuroprotection under stress conditions rather than to direct GABAergic modulation. Taken together, these findings suggest that the potential neuro-modulatory effects of GBP are primarily mediated through cholinergic and monoaminergic pathways, with only minor contributions from GABAergic activity. These mechanistic findings provide a biological context for previous clinical evidence linking both GBE and PS to improvements in memory performance, attentional control, and emotional regulation (6, 8, 9, 13, 15, 17, 24, 27–29, 32, 40), as modulation of cholinergic and monoaminergic pathways may support cognitive processing and mood regulation, while complementary antioxidant and neurotrophic mechanisms may contribute to stress resilience. The selection of cholinergic, monoaminergic, and GABAergic targets in the present study was guided by their well-established roles in cognitive performance, mood regulation, and stress resilience, as well as by prior experimental and clinical evidence indicating that both standardized GBEs and PS interact with these neurotransmitter systems. While the present work focused on these pathways, other mechanisms, including glutamatergic signaling and neuro-inflammatory processes, may also contribute to the overall neurocognitive effects of GBP and warrant investigation in future studies.

Oxidative stress and free radical damage are central mechanisms in neurodegenerative processes, cognitive decline, and mood disorders (40–42). In the present study, GBP demonstrated clear neuroprotective activity in cellular models, where it protected SH-SY5Y neuroblastoma cells from H_2_O_2_-induced oxidative stress. GBP treatment restored cell viability and reduced membrane damage, indicating preservation of neuronal integrity under oxidative challenge. These effects confirm its capacity to counteract oxidative stress–induced cytotoxicity, a mechanism directly relevant to the prevention of neuronal injury and functional decline.

Complementing these cellular findings, GBP also exhibited robust antioxidant activity across multiple cell-free assays, including DPPH, ABTS, FRAP, ORAC, HORAC, TAS and TPC. Together, these results indicate broad-spectrum radical-scavenging and reducing potential, reflecting the contribution of polyphenolic constituents to the extract. By neutralizing reactive oxygen species (ROS) and supporting redox homeostasis, GBP may mitigate one of the key pathophysiological drivers of cognitive decline and emotional disturbances such as low mood and impaired stress resilience. Although the antioxidant capacity of GB-based preparations has been previously reported, the use of multiple complementary cell-free antioxidant assays in the present study was used to mechanistically contextualize the observed neuroprotective effects under oxidative stress conditions in neuronal cells.

Building on these observations, we further examined whether GBP could influence BDNF expression under oxidative stress. BDNF is a critical mediator of synaptic plasticity and memory consolidation, and reduced levels have been implicated in both cognitive impairment and depressive symptoms (43). GBP preserved BDNF expression in SH-SY5Y cells exposed to H_2_O_2_, indicating a role in maintaining neurotrophic support under stress conditions. These findings are consistent with *in vivo* studies showing that standardized GBEs upregulate BDNF expression in the hippocampus, thereby promoting long-term memory persistence and enhancing learning outcomes (44, 45). By preserving both redox balance and BDNF expression, GBP may contribute to enhanced cognitive resilience and emotional well-being, complementing its neuro-modulatory effects.

The present mechanistic data provides a framework for interpreting clinical findings with the formulation of GBE and PS in Virtiva™ Plus. Controlled clinical trials have demonstrated improvements in memory, attention, and mental flexibility in healthy adults, as well as beneficial effects on emotional distress and physiological responses to prolonged exertion (27, 32). The current results offer mechanistic support for these outcomes, linking the observed cognitive and emotional benefits to cholinergic and monoaminergic modulation, antioxidant defense, and neurotrophic preservation. It is important to note that the concentrations employed in the present *in vitro* assays do not directly reflect achievable plasma or brain levels following oral supplementation. As is common in mechanistic *in vitro* studies of botanical preparations, higher concentrations are often required to overcome limitations related to cellular uptake, metabolic degradation, protein binding, and the absence of physiological accumulation processes (46–50). Accordingly, the concentrations used here were intended to explore biological pathways and mechanistic potential rather than to model *in vivo* exposure. These findings should therefore be interpreted as providing mechanistic plausibility supporting potential *in vivo* effects, which require confirmation in appropriately designed pharmacokinetic and clinical studies.

The inclusion of two active ingredients in GBP provides a rational explanation for the multifaceted biological effects observed in the present study. GB extract supplies flavonoids and terpenoids with antioxidants, anti-inflammatory, and cerebrovascular activity, while PS contributes to membrane fluidity, receptor function, and neurotransmitter release. PS may also optimize the incorporation of bioactive compounds into neuronal membranes. The complementary biological activities of these two agents likely account for the additive or synergistic benefits observed *in vitro*.

GBP potentially exerts mild but multi-targeted actions with the potential for fewer adverse effects, in respect to other conventional supports. By combining moderate enzyme inhibition with antioxidants and neurotrophic properties, it may provide a broader profile suited for preventive or adjuvant use. These characteristics are relevant not only to memory and executive function but also to emotional well-being and low mood. While nutraceuticals cannot replace established therapies in conditions such as AD or major depression, they may serve as useful adjuncts or preventive strategies to support cognition, stress resilience, and emotional health in otherwise health populations, not using pharmacological therapies. Safety is an essential consideration for nutraceutical strategies intended for long-term use. In this study, GBP showed no intrinsic cytotoxicity in SH-SY5Y cells across concentrations up to 1,500 μg/mL, consistent with the well-documented safety of both components in preclinical and clinical studies.

Taken together, these findings provide mechanistic evidence that GBP potentially supports neurotransmitter function, redox balance, and neurotrophic signaling processes that collectively underpin memory performance, executive function, and emotional well-being. These results add to the growing body of mechanistic evidence supporting the potential role of the supplementation of GB and PS as a natural strategy for maintaining cognitive health and emotional resilience.

This study has several limitations. The *in vitro* assays were designed to explore mechanistic interactions rather than to model physiological exposure levels. The complex composition of botanical extracts also precludes attribution of observed effects to individual constituents. These limitations are inherent to mechanistic food chemistry studies and highlight the need for future integrative approaches combining compositional analysis with *in vivo* validation.

## 5 Conclusion

This study provides mechanistic evidence supporting the biological plausibility of the association of GBE and PS in modulating neurotransmitter-related pathways, counteracting oxidative stress, and preserving neurotrophic support under adverse conditions. These complementary actions, possibly, acting additively and/or synergistically, underpin processes central to memory, executive function, and emotional well-being. To our knowledge, this is the first investigation to characterize the mechanistic basis of GBP, offering a biological rationale that supports its potential application as a nutraceutical strategy for cognitive and emotional health.

## Data Availability

The original contributions presented in the study are included in the article/supplementary material, further inquiries can be directed to the corresponding authors.
